# 6-Methyl­sulfanyl-4*H*-pyrimido[1,6-*a*]pyrimidin-4-one

**DOI:** 10.1107/S1600536809038562

**Published:** 2009-09-30

**Authors:** Quinhua Huang, Paul F. Richardson, Eugene Rui, Arnold L. Rheingold, Alex Yanovsky

**Affiliations:** a Pfizer Global Research and Development, La Jolla Labs, 10770 Science Center Drive, San Diego, CA 92121, USA; b Department of Chemistry and Biochemistry, University of California, San Diego, 9500 Gilman Drive, La Jolla, CA 92093, USA

## Abstract

Reaction of 2-(methyl­sulfan­yl)pyrimidin-4-amine with the 5-(methoxy­vinyl­idene) derivative of Meldrum’s acid and subsequent heating of the product in Dowtherm fluid yielded the title compound, C_8_H_7_N_3_OS, which was proven to contain a bicyclic 4*H*-pyrimido[1,6-*a*]pyrimidine system. All non-H atoms of the mol­ecule are coplanar within 0.15 Å. The bond-length distribution in the bicyclic core shows localization of the double bonds. The geometry of the intra­molecular S⋯O 1,5-contact [2.534 (2) Å] is consistent with the existence of an attractive inter­action.

## Related literature

For the structure of a compound with a similar bicyclic carbon–nitro­gen core, see: Olomucki *et al.* (1984 ▶). For statistical studies of the geometry of S⋯O inter­actions, see: Rosenfield *et al.* (1977 ▶); Iwaoka *et al.* (2002 ▶).
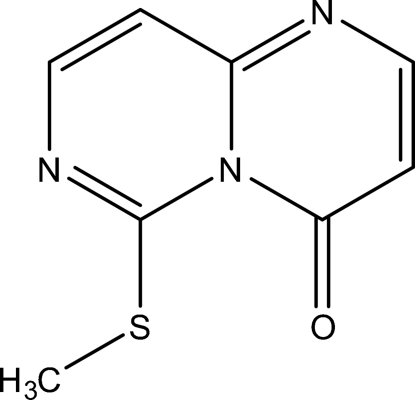


## Experimental

### 

#### Crystal data

C_8_H_7_N_3_OS
*M* *_r_* = 193.23
Monoclinic, 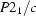

*a* = 9.7621 (8) Å
*b* = 4.1725 (3) Å
*c* = 20.4092 (16) Å
β = 100.106 (1)°
*V* = 818.42 (11) Å^3^
*Z* = 4
Mo *K*α radiation
μ = 0.35 mm^−1^
*T* = 123 K
0.48 × 0.14 × 0.08 mm

#### Data collection

Bruker APEXII CCD diffractometer
Absorption correction: multi-scan (*SADABS*; Bruker, 2001 ▶) *T*_min_ = 0.849, *T*_max_ = 0.972
6497 measured reflections
1498 independent reflections
1333 reflections with *I* > 2σ(*I*)
*R*_int_ = 0.022

#### Refinement

*R*[*F* ^2^ > 2σ(*F* ^2^)] = 0.035
*wR*(*F* ^2^) = 0.097
*S* = 1.13
1498 reflections
118 parameters
H-atom parameters constrained
Δρ_max_ = 0.43 e Å^−3^
Δρ_min_ = −0.19 e Å^−3^

### 

Data collection: *APEX2* (Bruker, 2004 ▶); cell refinement: *SAINT* (Bruker, 2004 ▶); data reduction: *SAINT*; program(s) used to solve structure: *SIR2004* (Burla *et al.*, 2005 ▶); program(s) used to refine structure: *SHELXL97* (Sheldrick, 2008 ▶); molecular graphics: *ORTEP-32* (Farrugia, 1997 ▶); software used to prepare material for publication: *WinGX* (Farrugia, 1999 ▶).

## Supplementary Material

Crystal structure: contains datablocks global, I. DOI: 10.1107/S1600536809038562/rz2363sup1.cif

Structure factors: contains datablocks I. DOI: 10.1107/S1600536809038562/rz2363Isup2.hkl

Additional supplementary materials:  crystallographic information; 3D view; checkCIF report

## Figures and Tables

**Fig. 1.**
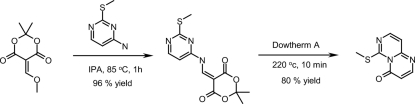
Synthesis of the title compound.

**Fig. 2.**
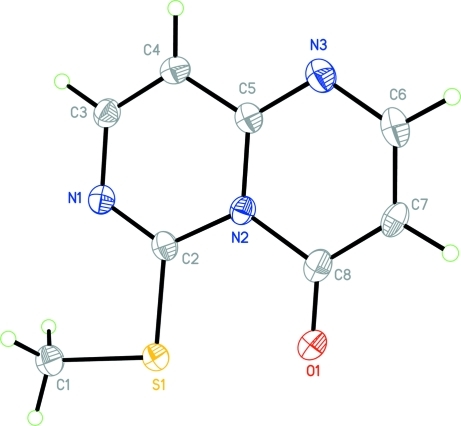
Molecular structure of the title compound, showing 50% probability displacement ellipsoids and atom numbering scheme. H atoms are drawn as circles with arbitrary small radius.

## supplementary crystallographic information

### Comment

The title compound was obtained by reaction of 2-(methylsulfanyl)pyrimidin-4-amine with 5-(methoxyvinylidene) derivative of Meldrum's acid and subsequent heating of the product in Dowtherm fluid (Fig. 1).

All non-hydrogen atoms of the molecule of the title compound (Fig. 2) are approximately coplanar; maximum deviation of the C1 atom from the least-squares plane is 0.143 (2) Å. The bond length values indicate localization of double bonds in the bicyclic core (N1–C2, 1.298 (3) Å; N3–C5, 1.308 (3) Å; C3–C4, 1.351 (3) Å; C6–C7, 1.355 (3) Å;), which is consistent with the fact that no resonance structures can be drawn. Surprisingly, to the best of our knowledge no other isolated bicyclic carbon-nitrogen system with the same positions of the N atoms was structurally studied. Similar bond lengths were observed in dihydropyrimido(1,2 - c)purine derivative, where analogous bicyclic core makes up a part of the tricyclic system (Olomucki *et al.*, 1984).

The S1···O1 distance [2.534 (2) Å] and the O1···S1—C1 angle [177.5 (1)°] are consistent with the existence of the intramolecular attractive interaction (Rosenfield *et al.*, 1977; Iwaoka *et al.*, 2002).

### Experimental

A mixture of (2-methylsulfanyl)pyrimidin-4-ylamine (5.0 g, 35 mmol, 1.0 eq), 5-(methoxyvinylidene) derivative of Meldrum's acid (9.23 g, 49.6 mmol, 1.4 eq), and 2-PrOH (100 ml) was refluxed at 85°C for 1 h to reach reaction completion. The resulting suspension was cooled to 25°C, filtered, and washed with EtOH to afford 10 g of the displacement product as a yellow solid in 96% yield. LC—MS (APCI, *M*+1) 296.0; ^1^H NMR (400 MHz, DMSO-d_6_) δ p.p.m. 1.69 (s, 6 H) 2.55 (s, 3 H) 7.43 (d, J = 5.54 Hz, 1 H) 8.57 (d, J = 5.54 Hz, 1 H) 9.20 (s, 1 H) 11.35 (s, 1 H).

The generated 5-[(2-methylsulfanyl)pyrimidin-4-ylaminovinylidene] derivative of Meldrum's acid (6.5 g, 22 mmol, 1.0 eq) was added portionwise to 130 ml of preheated Dowtherm A at 220°C (inner temperature). After being reacted at 220°C for 10 min, LC—MS indicated that reaction was complete. The reaction mixture was cooled to 25°C and diluted with 1 *L* heptane. The resulting suspension was left to stand overnight. The solid was filtered, washed with heptane, and dried under vacuum to afford 3.4 g of the target compound as a yellow solid in (80% yield). LC—MS (APCI, *M*+1) 194.0; ^1^H NMR (400 MHz, DMSO-d_6_) δ p.p.m. 2.40 (s, 3 H) 6.35 (d, J = 6.55 Hz, 1 H) 7.04 (d, J = 6.29 Hz, 1 H) 8.08 (t, J = 6.55 Hz, 2 H). The product was recrystallized from EtOAc/hexane to yield single crystals suitable for X-ray diffraction studies.

### Refinement

All H atoms were placed in geometrically calculated positions (C—H = 0.95 Å and 0.98 Å for aromatic and methyl H atoms respectively) and included in the refinement in riding motion approximation. The *U*_iso_(H) were set to 1.2*U*_eq_(C) or 1.5*U*_eq_(C) for methyl H atoms.

### Crystal data

**Table:**

C_8_H_7_N_3_OS	*F*(000) = 400
*M**_r_* = 193.23	*D*_x_ = 1.568 Mg m^−^^3^
Monoclinic, *P*2_1_/*c*	Mo *K*α radiation, λ = 0.71073 Å
Hall symbol: -P 2ybc	Cell parameters from 4134 reflections
*a* = 9.7621 (8) Å	θ = 3.2–25.4°
*b* = 4.1725 (3) Å	µ = 0.35 mm^−^^1^
*c* = 20.4092 (16) Å	*T* = 123 K
β = 100.106 (1)°	Blade, brown
*V* = 818.42 (11) Å^3^	0.48 × 0.14 × 0.08 mm
*Z* = 4	

### Data collection

**Table:**

Bruker APEXII CCD diffractometer	1498 independent reflections
Radiation source: fine-focus sealed tube	1333 reflections with *I* > 2σ(*I*)
graphite	*R*_int_ = 0.022
φ and ω scans	θ_max_ = 25.4°, θ_min_ = 2.1°
Absorption correction: multi-scan (*SADABS*; Bruker, 2001)	*h* = −11→11
*T*_min_ = 0.849, *T*_max_ = 0.972	*k* = −4→5
6497 measured reflections	*l* = −24→24

### Refinement

**Table:**

Refinement on *F*^2^	Primary atom site location: structure-invariant direct methods
Least-squares matrix: full	Secondary atom site location: difference Fourier map
*R*[*F*^2^ > 2σ(*F*^2^)] = 0.035	Hydrogen site location: inferred from neighbouring sites
*wR*(*F*^2^) = 0.097	H-atom parameters constrained
*S* = 1.13	*w* = 1/[σ^2^(*F*_o_^2^) + (0.045*P*)^2^ + 0.5615*P*] where *P* = (*F*_o_^2^ + 2*F*_c_^2^)/3
1498 reflections	(Δ/σ)_max_ = 0.001
118 parameters	Δρ_max_ = 0.43 e Å^−^^3^
0 restraints	Δρ_min_ = −0.19 e Å^−^^3^

### Special details

**Table:**

Geometry. All e.s.d.'s (except the e.s.d. in the dihedral angle between two l.s. planes) are estimated using the full covariance matrix. The cell e.s.d.'s are taken into account individually in the estimation of e.s.d.'s in distances, angles and torsion angles; correlations between e.s.d.'s in cell parameters are only used when they are defined by crystal symmetry. An approximate (isotropic) treatment of cell e.s.d.'s is used for estimating e.s.d.'s involving l.s. planes.
Refinement. Refinement of *F*^2^ against ALL reflections. The weighted *R*-factor *wR* and goodness of fit *S* are based on *F*^2^, conventional *R*-factors *R* are based on *F*, with *F* set to zero for negative *F*^2^. The threshold expression of *F*^2^ > σ(*F*^2^) is used only for calculating *R*-factors(gt) *etc*. and is not relevant to the choice of reflections for refinement. *R*-factors based on *F*^2^ are statistically about twice as large as those based on *F*, and *R*- factors based on ALL data will be even larger.

### Fractional atomic coordinates and isotropic or equivalent isotropic displacement parameters (Å^2^)

**Table:**

	*x*	*y*	*z*	*U*_iso_*/*U*_eq_	
S1	0.56563 (5)	0.91748 (12)	0.81678 (2)	0.02069 (19)	
O1	0.80408 (15)	0.9008 (4)	0.78390 (7)	0.0276 (4)	
N1	0.59517 (17)	0.5753 (4)	0.92701 (8)	0.0203 (4)	
N2	0.79659 (16)	0.5851 (4)	0.87620 (7)	0.0172 (4)	
N3	0.99408 (17)	0.2689 (4)	0.92351 (8)	0.0238 (4)	
C1	0.3990 (2)	0.9169 (5)	0.84302 (11)	0.0280 (5)	
H1A	0.3333	1.0474	0.8123	0.042*	
H1B	0.4089	1.0064	0.8880	0.042*	
H1C	0.3642	0.6966	0.8431	0.042*	
C2	0.65797 (19)	0.6714 (5)	0.87950 (9)	0.0183 (4)	
C3	0.6652 (2)	0.3762 (5)	0.97497 (10)	0.0209 (4)	
H3B	0.6193	0.3094	1.0100	0.025*	
C4	0.7959 (2)	0.2700 (5)	0.97507 (9)	0.0203 (4)	
H4A	0.8392	0.1284	1.0089	0.024*	
C5	0.8678 (2)	0.3717 (5)	0.92427 (9)	0.0186 (4)	
C6	1.0583 (2)	0.3766 (5)	0.87347 (11)	0.0254 (5)	
H6A	1.1495	0.3010	0.8719	0.030*	
C7	0.9993 (2)	0.5853 (5)	0.82589 (9)	0.0199 (4)	
H7A	1.0506	0.6512	0.7927	0.024*	
C8	0.8652 (2)	0.7063 (5)	0.82424 (9)	0.0219 (4)	

### Atomic displacement parameters (Å^2^)

**Table:**

	*U*^11^	*U*^22^	*U*^33^	*U*^12^	*U*^13^	*U*^23^
S1	0.0177 (3)	0.0225 (3)	0.0219 (3)	0.00111 (19)	0.0038 (2)	0.0028 (2)
O1	0.0237 (8)	0.0363 (9)	0.0241 (7)	0.0002 (7)	0.0079 (6)	0.0095 (7)
N1	0.0196 (8)	0.0199 (9)	0.0220 (8)	−0.0009 (7)	0.0053 (7)	−0.0002 (7)
N2	0.0184 (8)	0.0176 (8)	0.0164 (8)	−0.0021 (6)	0.0050 (6)	−0.0017 (6)
N3	0.0199 (8)	0.0240 (9)	0.0278 (9)	0.0015 (7)	0.0047 (7)	−0.0014 (8)
C1	0.0201 (11)	0.0335 (12)	0.0312 (11)	0.0032 (9)	0.0068 (9)	0.0059 (10)
C2	0.0169 (9)	0.0178 (9)	0.0201 (10)	−0.0015 (8)	0.0026 (8)	−0.0037 (8)
C3	0.0211 (10)	0.0232 (10)	0.0190 (10)	−0.0025 (8)	0.0052 (8)	0.0004 (8)
C4	0.0233 (10)	0.0189 (10)	0.0184 (10)	−0.0007 (8)	0.0026 (8)	0.0006 (8)
C5	0.0179 (10)	0.0173 (9)	0.0200 (10)	−0.0016 (8)	0.0022 (8)	−0.0041 (8)
C6	0.0180 (10)	0.0264 (11)	0.0326 (11)	−0.0005 (9)	0.0068 (9)	−0.0070 (9)
C7	0.0253 (11)	0.0186 (10)	0.0183 (10)	−0.0042 (8)	0.0108 (8)	−0.0040 (8)
C8	0.0234 (10)	0.0245 (11)	0.0186 (10)	−0.0066 (9)	0.0059 (8)	−0.0026 (9)

### Geometric parameters (Å, °)

**Table:**

S1—C2	1.761 (2)	C1—H1B	0.9800
S1—C1	1.799 (2)	C1—H1C	0.9800
O1—C8	1.233 (2)	C3—C4	1.351 (3)
N1—C2	1.298 (3)	C3—H3B	0.9500
N1—C3	1.371 (3)	C4—C5	1.415 (3)
N2—C2	1.413 (2)	C4—H4A	0.9500
N2—C5	1.414 (2)	C6—C7	1.355 (3)
N2—C8	1.442 (2)	C6—H6A	0.9500
N3—C5	1.308 (3)	C7—C8	1.398 (3)
N3—C6	1.365 (3)	C7—H7A	0.9500
C1—H1A	0.9800		
			
C2—S1—C1	99.03 (9)	N1—C3—H3B	118.2
C2—N1—C3	118.60 (17)	C3—C4—C5	119.37 (18)
C2—N2—C5	119.08 (16)	C3—C4—H4A	120.3
C2—N2—C8	121.20 (16)	C5—C4—H4A	120.3
C5—N2—C8	119.70 (16)	N3—C5—N2	123.26 (18)
C5—N3—C6	117.29 (18)	N3—C5—C4	119.98 (18)
S1—C1—H1A	109.5	N2—C5—C4	116.76 (17)
S1—C1—H1B	109.5	C7—C6—N3	123.66 (19)
H1A—C1—H1B	109.5	C7—C6—H6A	118.2
S1—C1—H1C	109.5	N3—C6—H6A	118.2
H1A—C1—H1C	109.5	C6—C7—C8	121.85 (18)
H1B—C1—H1C	109.5	C6—C7—H7A	119.1
N1—C2—N2	122.57 (17)	C8—C7—H7A	119.1
N1—C2—S1	118.33 (15)	O1—C8—C7	126.55 (18)
N2—C2—S1	119.10 (14)	O1—C8—N2	119.26 (18)
C4—C3—N1	123.53 (18)	C7—C8—N2	114.19 (17)
C4—C3—H3B	118.2		
